# Electronic Booster PEDOT:PSS-Enriched Guar Gum as Eco-Friendly Gel Electrolyte for Supercapacitor

**DOI:** 10.1021/acsomega.4c00786

**Published:** 2024-05-28

**Authors:** Sumana Vittal Subrahmanya, Sudhakar Narahari Yethadka, Nagaraja G K

**Affiliations:** † Department of Chemistry, Srinivas Institute of Technology, Valachil 574143, India; ‡ Department of Chemistry, Manipal Institute of Technology, Manipal Academy of Higher Education, Manipal 576104, India; § Department of Chemistry, Mangalore University, Mangalagangotri, Konaje 574199, India

## Abstract

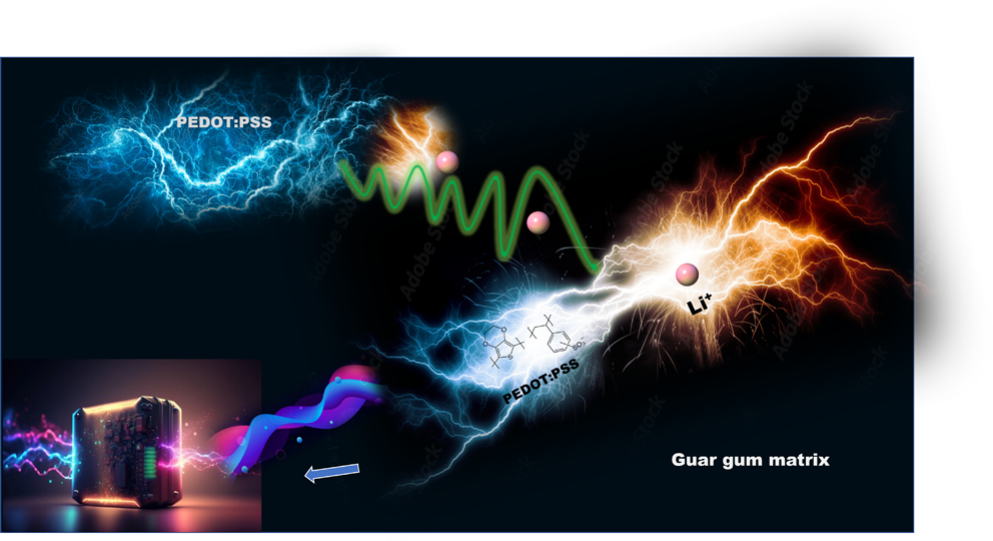

Supercapacitors based on biobased materials have been regarded as alternative portable energy storage technology for wearable or flexible electronics. Herein, we construct a unique electronic booster-imbedded biopolymer electrolyte with enhanced power density and long cycling life quasi-solid-state supercapacitor using poly(3,4-ethylenedioxythiophene):poly(styrenesulfonate) PEDOT:PSS/guar gum (GG). The eco-friendly 2% (v/v) PEDOT:PSS in the GG matrix doped with 0.05 wt % of lithium perchlorate (LiClO_4_) was highly flexible and showed an ionic conductivity of 10^–2^ S cm^–1^ at 323 K. The surface morphology showed unique potential wells of PEDOT:PSS boosting the nonconductive GG and interaction with activated carbon-based electrodes. As a result, a specific capacitance of 141 F g^−1^ at 5 mV s^–1^ was observed. The cyclic stability was 98% even after 1000 charge–discharge study cycles. To the best of our knowledge, this is the first work demonstrating a high-performance supercapacitor with conducting polymer-boosted guar gum as the polymer gel electrolyte, and it provides scope for understanding further stability testing and the interaction mechanism within the polymer matrix.

## 1 Introduction

The supercapacitor is an energy storage device that produces energy and power simultaneously for a sudden requirement. The energy can be used through a faradaic process, electrochemical double-layer charging, or sometimes synergistic outcomes based on both. The increase in energy requirement has jumped many times in the past decade, with more emphasis on sustainable development goals (SDGs). Affordable and clean energy is one of the goals of SDG. There is a paradigm shift in the research that incorporates eco-friendly materials without compromising the basic property requirement of an energy device. Although the presently commercialized energy storage devices can meet particular demands to quite an extent because of extensive research in this field, they lack solutions to what can be done when their cycle life is over. So, a set of researchers are working on solid-state energy devices that incorporate eco-friendly materials such as biodegradable polymers,^1^ hydrogel-based,^2^ and activated carbon from natural sources.^3^ The biodegradable polymer electrolyte provides flexibility and no risk of leakage, acts as a separator, and has sufficient ionic conductivity. The gel polymer electrolyte usually has high ionic conductivity compared to the blend or solid polymer electrolyte, as it provides more of the jelly matrix for the movement of ions. Moreover, it reduces the resistance at the electrode–electrolyte interface area.^4^

Guar gum (GG) is a polysaccharide extracted from guar beans and is mainly used as a thickener and stabilizer in food items. The water retention property is also suitable and can be used in energy storage devices.^5^ GG, a biodegradable polymer, can be an example of clean energy in SDG, a renewable source, leading to future investments.^6^ In our previous work, a tubular array of GG exhibited high energy density with an ionic conductivity of 10^–3^ S cm^–1^. GG polymer electrolyte was plasticized with glycerol to achieve this ionic conductivity.^7^ Furthermore, GG/poly(vinyl alcohol) (PVA) blend polymer electrolytes and uniquely equipped activated carbon (AC) prepared from areca nuts were prepared. A relatively higher specific capacitance was observed than in plasticized GG polymer electrolytes. The binder for AC used was also GG, which reduced the resistance at an electrode/electrolyte interface.^8^ Hence, in this study, we introduced a conducting polymer known for its electrical conductivity, i.e., poly(3,4-ethylenedioxythiophene):polystyrenesulfonate (PEDOT:PSS). It has excellent electrochemical properties, good dispersion, and is stretchable.^9^ PEDOT:PSS lacks mechanical strength and is not easy to use directly as a polymer electrolyte in supercapacitors.^10^ Conducting polymer as a composite or dopant must not affect a polymer host’s biodegradability and mechanical strength. The benefits of softness, bendability, high electrical conductivity, and good stability in the polymer matrix are maintained even though the active dopant PEDOT:PSS is incorporated in it.^11^ Due to interconnected networks by PEDOT:PSS in the polymer matrix, other than energy devices, a next-generation PEDOT:PSS nanofibril-doped hydrogel bioelectronic is also of great interest.^12^ In another study, ionic liquid (IL) 1-butyl-3-methylimidazolium chloride (BMIMCl) provided a reduced vapor pressure, and interestingly, introducing PEDOT:PSS produced a redox behavior with high ionic conductivities of 10^–2^ S cm^–1^.^13^ A transparent PEDOT-based device produced power/energy densities with an areal capacitance of 1.32 mF cm^–2^, which is remarkably more significant than most of the reported flexible and transparent conducting polymer films.^14^ The ease of preparation of polymer electrolytes using a PEDOT:PSS suspension at room temperature followed by a freeze-drying process as a channel material for fabricating organic bioelectronic devices is also gaining interest. Furthermore, by adding graphene-PEDOT to PVA, a hydrogel fiber is developed. It has excellent flexibility and unique bicontinuous networks. The specific capacitance was found to be 281 F g^–1^ at 25 °C. The polymer electrolyte showed antifreeze properties, mechanical performance, and high ionic conduction.^15^ This work prepares a unique combination of GG and PEDOT:PSS as high ionic and electric conducting electrolytes for supercapacitors. The electrochemical impedance spectroscopic studies were performed to understand the ionic conductivity of the GG/PEDOT:PSS electrolyte.

## 2 Experimental Section

### 2.1 Material Preparation

Guar gum (medium molecular weight) and PEDOT:PSS (3–4%) were purchased from Merck. Lithium perchlorate (Merck) was dried before use. 3% (wt/v) of GG was stirred until gelation and was used as a stock solution. Briefly, the GG powder was taken in a 100 mL beaker, and double-distilled water was added slowly while stirring in a magnetic stirrer at 50 °C. The lumps were crushed using a glass rod and gently heated in the beaker to reduce the volume to a thick jelly appearance. 0.5, 1, 1.5, and 2% (v/v) of PEDOT:PSS suspensions were directly added to a Petri dish containing sufficient GG solution and labeled A1, A2, A3, and A4, respectively. The solution was stirred mildly for 40 h at 40 °C for uniform dispersion by maintaining a solvent level of 3/fourth of the Petri dish. The high content of the PEDOT:PSS-containing solution turned into a pale blue solution. The added LiClO_4_ concentration was optimized to 0.05 wt % from 0.01 to 0.05 wt % and mixed thoroughly for 4 h. The Petri plates were kept in a hot air oven at 60 °C until a smooth, free-standing jelly film of varying thickness from 1 to 2 mm was obtained.

### 2.2 Characterization

Attenuated total reflectance-Fourier transform infrared (ATR-FTIR) studies were performed between 400 and 4000 cm^–1^ wavenumbers in the transmittance mode with a resolution of 4 cm^–1^ using Nicolet Thermo Scientific’s iZ10 FTIR. The deep-frozen dry gel polymer electrolyte (GPE) was kept in the nitrogen atmosphere and then subjected to a high vacuum using scanning electron microscope (SEM), ZEISS EVO18. The films were cut into 2 × 2 cm^2^ pieces and sandwiched between stainless steel electrodes. The bulk ionic conductivities (σ) were studied by using alternating current (AC) impedance spectroscopy at a frequency range between 1 MHz and 100 mHz using a small-amplitude AC signal of 10 mV in the Biologic SP50e instrument.

### 2.3 Fabrication of Supercapacitor

The activated carbon (AC) electrode was prepared, as previously reported in the article.^8^ The two best electrodes were used for supercapacitor fabrication. The GPE was sandwiched between two AC-coated electrodes and sealed in a plastic-coated aluminum-recycled tetra pack under the nitrogen atmosphere to avoid oxidation of Li salts during fabrication. The electrochemical characterizations, such as cyclic voltammetry (CV), AC impedance, galvanostatic charge/discharge studies, and cyclic stability, were studied.

## 3 Results and Discussion

### 3.1 FTIR Studies of GG/PEDOT:PSS Films

**Figure 1:**
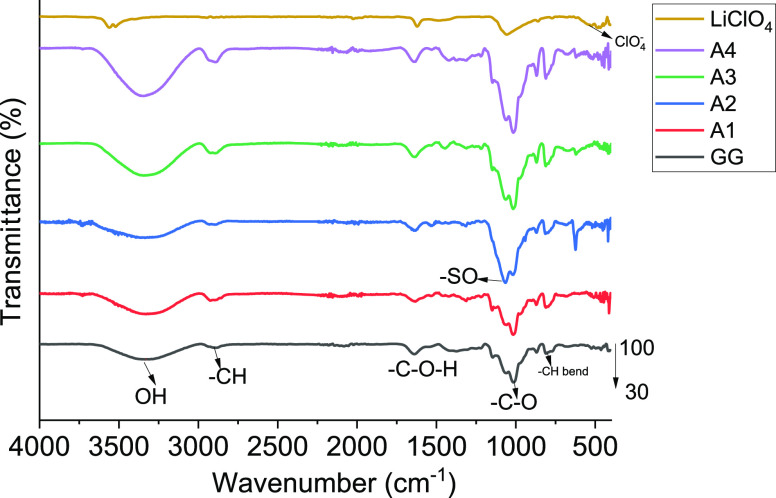
Comparison of ATR-FTIR spectra of different samples of GPEs.

The interaction of GG/PEDOT:PSS is studied by using ATR-FTIR studies (Figure 1). The pure GG shows a broad peak at 3350 cm^–1^ due to the O–H stretching, and on the addition of lithium salt, it has shifted to 3401 cm^–1^. In the FTIR spectrum of LiClO_4_, the characteristic peak at 630 cm^–1^ disappeared, and a new peak was observed for the doped samples at 681 cm^–1^. With the increase in salt concentration, this peak intensified, indicating further interaction of GG with added LiClO_4_. The presence of GG in all of the samples from A1 to A4 seems to be the same, as there are no significant changes in the position of peaks of OH stretching and CH stretching at 3401 and 2934 cm^–1^, respectively.^16^ However, with increased PEDOT:PSS, the characteristic transmittance peaks at 2912 and 1500 cm^–1^ attributed to C–H stretching and C–O–H bending, respectively, slightly broadened. These shifts and broadening indicate that hydrogen bonding between PEDOT:PSS and GG has formed. The peaks at 1071 and 1031 cm^–1^ due to the C–O stretching vibration and the S–O symmetrical bond of the sulfonate group from PSS, respectively, became predominant with increased PEDOT:PSS in the GPE, indicating improved interaction with Li ions and GG polymer matrix. Furthermore, the peak at 890 cm^–1^ is due to CH bond rocking.^17^ Along with the interaction peaks between GG and PEDOT:PSS, another peak is due to the Li salt in the matrix at 1220 cm^–1^. Hence, the GPE shows the interaction between GG and PEDOT:PSS, which helps improve the stability during charging and discharging. In summary, the weakening of CH-bonded sulfate group bands upon an increase in the PEDOT:PSS concentration shows the interaction of GG with PEDOT:PSS. Moreover, the SO^3–^ group attracts Li ions upon increased salt concentration, indicating that PEDOT:PSS boosts Li ions’ movement and connects to GG to enhance its overall conductivity electronically.

### 3.2 SEM Analysis

**Figure 2:**
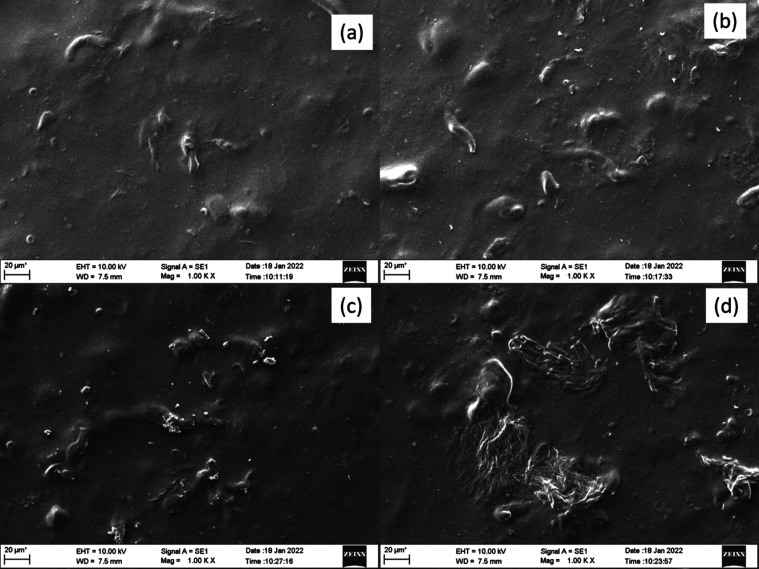
SEM images of (a) A1, (b) A2, (c) A3, and (d) A4.

The SEM images of A1, A2, A3, and A4 are shown in Figure 2a–d, respectively. In Figure 2a, the surface morphology is smooth, without any salt accumulation or phase separation. Uniform distribution of PEDOT:PSS is observed. Figure 2b shows that with increased PEDOT:PSS, the surface shows dispersed tubular phases. In Figure 2c, the network of PEDOT:PSS starts to be evident on the surface. In Figure 2d, clusters of PEDOT:PSS with tubular projections are distributed all along the surface of GPE. The network of PEDOT:PSS helps to enhance ionic conductivity by providing transport channels for Li ions. Furthermore, no porous surfaces are observed, which means the GPE has good solvent retention properties. This will help to maintain the semisolid nature during the higher number of charge/discharge cycles.

### 3.3 Ion Conductivity Studies

**Figure 3:**
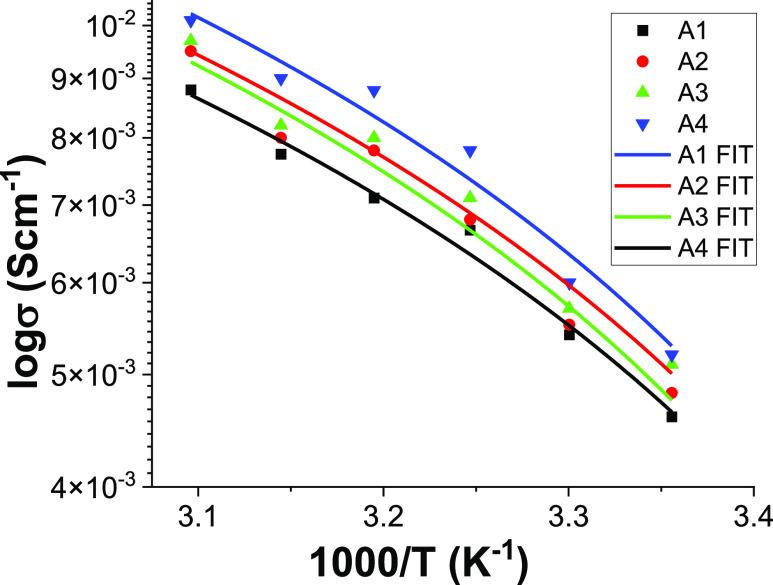
Variations of conductivities of different blend compositions of BPE at different temperatures.

Ionic conductivities calculated from the AC impedance data are shown in Figure 3. With temperature variation, the plot showed a nonlinear pattern. This indicates that the GPE has neither a cluster of Li ions nor phase separation but only ionic movement through a smooth matrix. Interestingly, the ionic conductivity was 1.1 × 10^–2^ S cm^–1^ at 323 K for the A4 sample, while 8.8 × 10^–3^ S cm^–1^ at 323 K for the A1 sample. The increase in temperature enhanced the charge carrier mobility due to increased segmental motion.^18,19^ Moreover, the salt in the polymer electrolyte may vibrate at high amplitude and increase its free ion concentration, contributing to conductivity.^19,20^ The presence of PEDOT:PSS promotes electric conductivity due to the negatively charged groups in PSS such as SO^3–^ or SO_3_H groups and, hence, boosts Li ionic conductivity by providing the electrostatic interaction in the medium. In the presence of PEDOT:PSS, the high temperature increases the polymer’s segmental motion, allowing Li ions to hop on the chains. Hence, there is a slight deviation from the straight line.

### 3.4 Supercapacitor Studies

**Figure 4:**
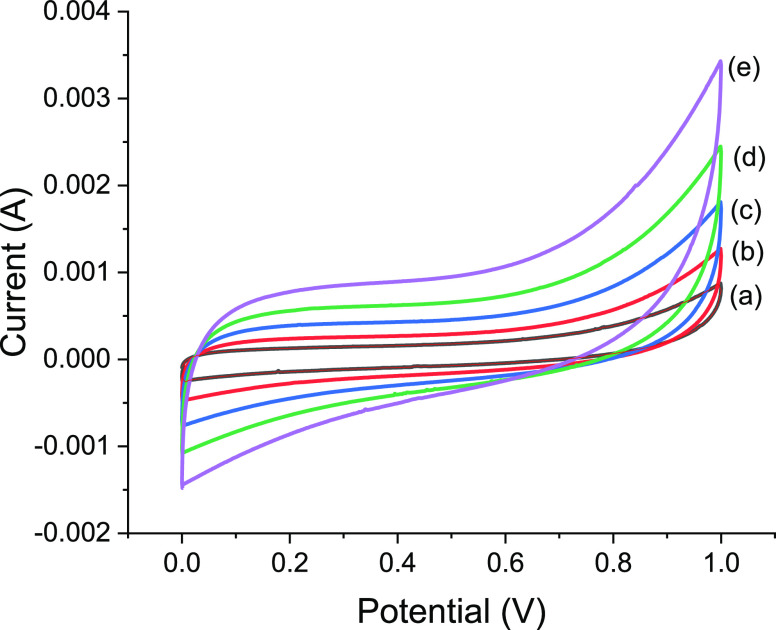
CVs of supercapacitor containing GPE at different scan rates: (a) 5 mV s^–1^, (b) 10 mV s^–1^, (c) 20 mV s^–1^, (d) 30 mV s^–1^, and (e) 40 mV s^–1^.

Cyclic voltammetry studies were performed using fabricated symmetric supercapacitors at different scan rates using the A4 sample, which showed the highest ionic conductivity (Figure 4). A typical double-layer capacitance-based rectangular pattern was observed. The specific capacitance was calculated using the equation *C* = (2Δ*I*)/(Δ*V × m*), where Δ*V* is the voltage scan rate, *m* is the mass per electrode, Δ*I* is the average current, and *C* is the specific capacitance.^1^ The change in the scan rate did not affect the CV pattern. Hence, the GPE exhibits good stability and reversibility during voltage fluctuation. The specific capacitance of A4 containing a supercapacitor showed 141, 81, 45, 34, and 30 F g^–1^ at 5, 10, 20, 30, and 40 mV s^–1^, respectively. This high capacitance is mainly due to the contribution of polarization at the electrode/electrolyte interface.

**Figure 5:**
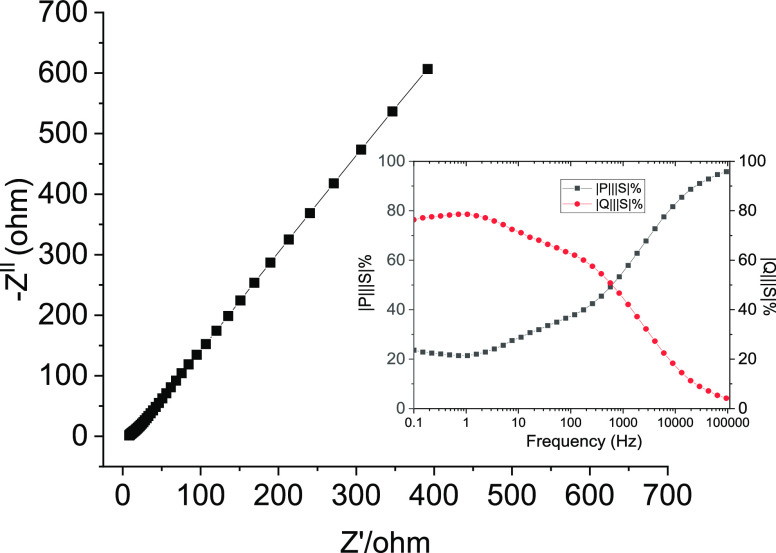
AC impedance plot of supercapacitor [inset: plots of the normalized reactive power |*Q*|/|*S*|% and active power |*P*|/|*S*|% versus frequency].

The AC impedance study was performed to determine the *C*_dl_, the double-layer capacitance. The Nyquist plot is shown in Figure 5. Using this resistance at high frequency, the value of *C*_dl_, the double-layer capacitance, has been determined from the high-frequency region of the impedance spectrum using the equation *Z*″ = 1/(2 × π × *f* × *C*). Herein, *Z*″ (imaginary impedance) is plotted against 1/*f* (reciprocal of the frequency), and the slope of the linear portion on the plot at the low-frequency end is used to derive the capacitance. The *C*_dl_ was found to be 426 mF cm^–2^. The pattern shows that the imaginary part of impedance sharply increases at a lower frequency, confirming the capacitive behavior of the GPE electrolyte. The time constant (τ) was calculated to study the transition for the supercapacitor between resistive behavior for frequencies higher than 1/τ and capacitive behavior for frequencies lower than 1/τ. Using the AC impedance data, the normalized reactive power |*Q*|/|*S*|% and active power |*P*|/|*S*|% versus frequency plot (inset, Figure 5) were plotted, and the time constant was found to be 0.3 s. This indicates that the present supercapacitors work efficiently at lower frequencies.

**Figure 6:**
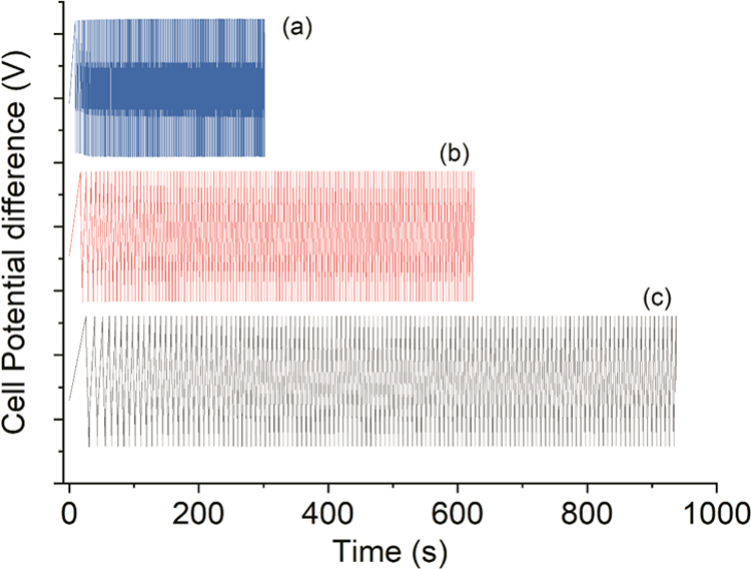
GCD curves at (a) 0.5 mA cm^–1^, (b) 1 mA cm^–1^, and (c) 1.5 mA cm^–1^.

**Figure 7:**
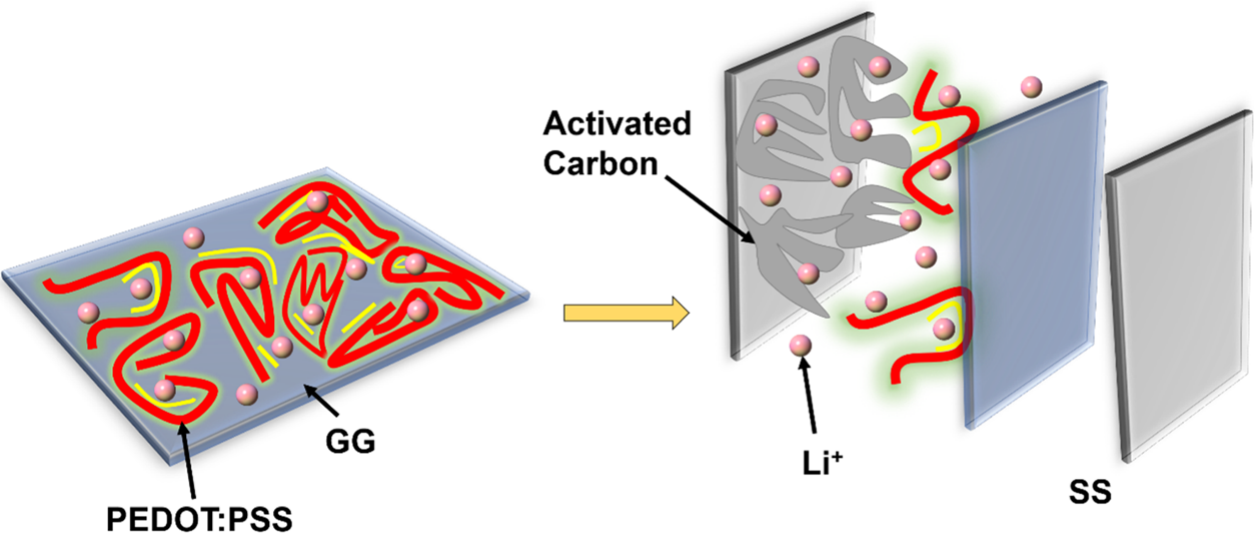
Probable interaction of Li ions with AC and PEDOT:PSS/GG polymer electrolyte.

The cyclic stability of supercapacitor at a constant current, i.e., galvanostatic charge–discharge (GCD) studies, was performed between 0 and 1 V potential window at 0.5, 1, and 1.5 mA cm^–1^ (Figure 6). The rapid charge–discharge was observed up to a studied range of 1000 cycles. A capacitive type of pattern in the form of a triangle was observed with a small *iR* drop (*i* means current, and *R* is the resistance). The discharge capacitance (*C*_d_) was calculated from the literature,^21^ and the values of charge–discharge cycles measured at the lowest current, 0.5 mA cm^–1^, were found to be 475 and 364 mF cm^–2^ for the initial and 1000th cycles, respectively, with a Coulombic efficiency of 98%. The equivalent series resistance (ESR) was 41 Ω with an *iR* drop of 0.02 V. The specific energy and specific power densities were 43.79 W kg^–1^ and 186.2 Wh kg^–1^, respectively. The presence of PEDOT:PSS enhanced the charging and discharging as it supports electrical conductance, and GG provides a jelly medium for the transport of ions. High power density is mainly observed due to the dual characteristics of the GPE. Figure 7 shows the most probable mechanism of interaction of Li ions with activated PEDOT:PSS, which energies the nonconductive GG matrix and helps to hold them for a longer time, enhancing the power density. Nevertheless, the energy density is relatively low, which may be due to a lack of hindrance for the ions while moving toward the electrode. The potential wells of PEDOT:PSS interact further with activated carbon, thereby improving the accessibility of ions at the electrode/electrolyte interface. Moreover, the polymer segments inside potential wells undergo constant contraction and expansion during charge/discharge, avoiding the formation of dendrites of the Li salt. Table 1 compares the supercapacitor parameters of the present work with those of similar reported articles.

**Table 1:** Comparison of Specific Capacitance with Biopolymer Electrolyte-Based Supercapacitors

electrolyte	specific capacitance (F g^-1^)	refs
PCL and (1-ethyl-3-methylimidazolium trifluoromethane-sulfonate) (EMITf)	170	(13)
rGO/SWNT/PCL	52.5	(14)
PCL/ammonium thiocyanate and ethylene carbonate	42	(15)
Xanthan gum/Na_2_SO_4_	347	(16)
Polypyrrole/Gum Arabic	168	(17)
GG/PEDOT:PSS/LiClO_4_	141	present work

## 4 Conclusions

A unique combination of electric and ionic conducting biodegradable GPEs was prepared by using PEDOT:PSS and Li salt. Interestingly, it showed a high ionic conductivity of around 10^–2^ S cm^–1^ at 323 K. As revealed in SEM images, the tubular clusters of PEDOT:PSS provided channels for the transport of Li ions in the GG matrix. The FTIR studies showed the interaction of GG with PEDOT:PSS and LiClO_4_. The sulfate group acted as a booster to attract Li ions, thereby providing the dual role of electronic and ionic conductivity enhancers. Fabricated supercapacitors using A4, the highest conducting electrolyte, showed a relatively high specific capacitance and power density but low energy density. The discharge capacitance was relatively stable for 1000 cycles, which indicated the stability of the prepared polymer electrolyte. This is mainly due to phase-separation-free channels for electric conduction and ionic conduction in the GG matrix. Hence, this paper provides a new challenge of incorporating conducting polymers that are electrically active and exhibit the same properties as metallic-doped GPEs.
